# Plasma Homocysteine and Polymorphisms of Genes Involved in Folate Metabolism Correlate with *DNMT1* Gene Methylation Levels

**DOI:** 10.3390/metabo9120298

**Published:** 2019-12-07

**Authors:** Fabio Coppedè, Andrea Stoccoro, Pierpaola Tannorella, Lucia Migliore

**Affiliations:** Department of Translational Research and of New Surgical and Medical Technologies, University of Pisa, Via Roma 55, 56126 Pisa, Italy; andrea.stoccoro@unipi.it (A.S.); pierpaola.tannorella@istituto-besta.it (P.T.); lucia.migliore@med.unipi.it (L.M.)

**Keywords:** DNMT1, DNA methylation, DNA methyltransferase, epigenetics, folate, homocysteine, polymorphisms, *MTR*, *TYMS*

## Abstract

DNA methyltransferase 1 (DNMT1) is responsible for the maintenance of DNA methylation patterns during cell division. Several human diseases are characterized by impaired *DNMT1* gene methylation, but less is known about the factors that regulate *DNMT1* promoter methylation levels. Dietary folates and related B-vitamins are essential micronutrients for DNA methylation processes, and we performed the present study to investigate the contribution of circulating folate, vitamin B12, homocysteine, and common polymorphisms in folate pathway genes to the *DNMT1* gene methylation levels. We investigated *DNMT1* gene methylation levels in peripheral blood DNA samples from 215 healthy individuals. All the DNA samples were genotyped for *MTHFR* 677C > T (rs1801133) and 1298A > C (rs1801131), *MTRR* 66A > G (rs1801394), *MTR* 2756A > G (rs1805087), *SLC19A1* (*RFC1*) 80G > A (rs1051266), *TYMS* 28-bp tandem repeats (rs34743033) and 1494 6-bp insertion/deletion (indel) (rs34489327), *DNMT3A* -448A > G (rs1550117), and *DNMT3B* -149C > T (rs2424913) polymorphisms. Circulating homocysteine, folate, and vitamin B12 levels were available from 158 of the recruited individuals. We observed an inverse correlation between plasma homocysteine and *DNMT1* methylation levels. Furthermore, both *MTR* rs1805087 and *TYMS* rs34743033 polymorphisms showed a statistically significant effect on *DNMT1* methylation levels. The present study revealed several correlations between the folate metabolic pathway and *DNMT1* promoter methylation that could be of relevance for those disorders characterized by altered DNA methylation.

## 1. Introduction

**Figure 1:**
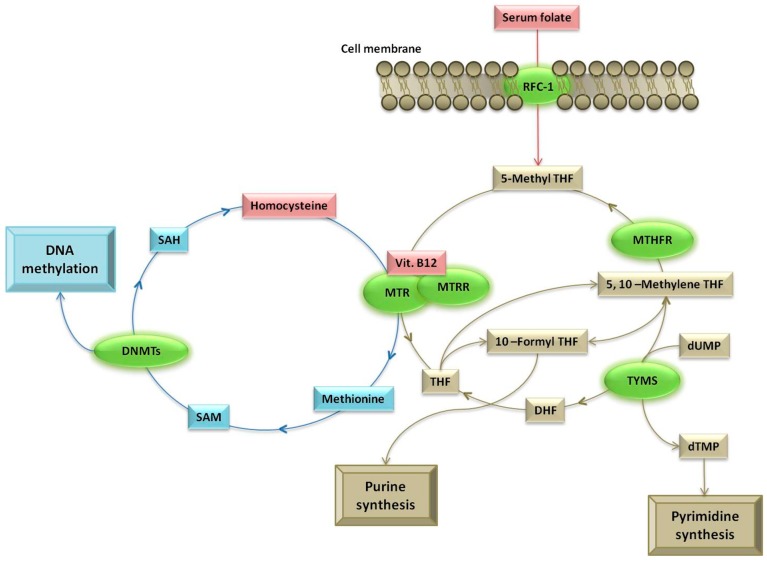
Simplified overview of the folate (light brown color) and methionine (heavenly color) cycles in one-carbon metabolism, adapted from Reference [4]. The diagram illustrates the enzymes (green color) whose polymorphisms were investigated in this article, and their metabolites or cofactors (those analyzed in the present study are in red). Enzymes: DNMTs, DNA methyltransferases; MTHFR, methylenetetrahydrofolate reductase; MTR, methionine synthase; MTRR, methionine synthase reductase; RFC1, reduced folate carrier 1; TYMS, thymidilate synthase. Metabolites: DHF, dihydrofolate; THF, tetrahydrofolate; dTMP, deoxythymidine monophosphate; dUMP, deoxyuridine monophosphate; SAH, *S*-adenosylhomocysteine; SAM, *S*-adenosylmethionine.

DNA methylation is one of the most investigated epigenetic mechanisms that tightly regulate gene transcription and expression levels, and it consists of the addition of a methyl group to the DNA, mediated by a class of enzymes called DNA methyltransferases (DNMTs) [1]. DNA methylation usually occurs on cytosine, forming 5-methylcytosine (5-mC) in a cytosine–phosphate–guanine (CpG) dinucleotide context, and CpG methylation in the promoter region of a gene represses gene expression [2]. The human genome encodes five DNMTs, namely, DNMT1, DNMT2, DNMT3A, DNMT3B, and DNMTL [3]. Among them, DNMT1 is the “maintenance” DNMT that maintains DNA methylation patterns during cell division, while DNMT3A and DNMT3B are responsible for the establishment of de novo DNA methylation patterns during embryogenesis. DNMT3L is not an active DNMT, but an important cofactor for DNMT3 enzymatic activity, whilst DNMT2 is likely an ancient DNMT that switched its substrate from DNA to RNA [3]. Micronutrients such as folate, vitamin B12, and other B-group vitamins contribute to DNA methylation as methyl donors and cofactors (Figure 1), and the status of these nutrients correlates with both global and gene-specific methylation levels [4]. The “core” parts of one-carbon metabolism are the folate and methionine cycles, a set of interconnected pathways required for the synthesis of *S*-adenosylmethionine (SAM), the main intracellular methylating agent. Indeed, DNMTs catalyze the transfer of the methyl group from SAM to the DNA (Figure 1).

As discussed above, DNMT1 is the main enzyme responsible for the maintenance of DNA methylation during cell division, and previous studies revealed the existence of a CpG island in the promoter/5′ untranslated region (5′UTR) of the *DNMT1* gene itself, whose methylation levels regulate *DNMT1* gene expression in both healthy and pathological tissues [5,6,7,8,9,10,11,12]. In this regard, increased *DNMT1* promoter methylation resulting in decreased gene expression levels was observed in blood DNA of individuals with ankylosing spondylitis, a chronic inflammatory autoimmune disease [5]. Similarly, a decreased *DNMT1* expression was observed in blood DNA of individuals with Kawasaki disease, an acute vasculitis syndrome [6], whilst increased *DNMT1* expression was observed in blood DNA from patients with psoriasis [7]. Changes in *DNMT1* promoter methylation were also observed in peripheral blood DNA samples from patients with acute lymphoblastic leukemia (ALL) and were linked to the *DNMT1* expression pattern in the patients [8]. Furthermore, DNMT1 expression levels in white blood cells of healthy individuals showed a large inter-individual variability [9]. Hypermethylation of the *Dnmt1* promoter was also responsible for reduced gene expression levels in various tissues of animal models of asthma [10]. The investigation of *DNMT1* methylation, expression, and protein levels in solid tumors showed a large variability according to the investigated cell line or to the cancer subtype [9,11,12,13].

Little is still known concerning genetic, dietary, or environmental factors linked to changes in *DNMT1* methylation levels. In this regard, a previous investigation in peripheral blood DNA samples of Alzheimer’s disease (AD) individuals suggested that the promoter methylation levels of several genes, including *DNMT1*, could be linked to circulating markers of folate metabolism, and particularly to plasma homocysteine (Hcy) levels [14].

The present study was performed to better address the contribution of folate metabolism to *DNMT1* gene methylation levels. We collected blood samples from 215 healthy individuals, investigated *DNMT1* methylation levels in the extracted DNA, and searched for correlation between *DNMT1* promoter methylation levels and circulating folate, Hcy, or vitamin B12 levels. In addition, the major polymorphisms on folate-pathway genes, such as methylenetetrahydrofolate reductase (*MTHFR*) 677C > T (rs1801133) and 1298A > C (rs1801131), methionine synthase (*MTR*) 2756A > G (rs1805087), methionine synthase reductase (*MTRR*) 66A > G (rs1801394), thymidilate synthase (*TYMS*) 28-bp tandem repeat (rs34743033) and 1494 insertion/deletion (indel) (rs34489327), and reduced folate carrier (*SLC19A1* or *RFC1*) 80A > G (rs1051266), as well as both *DNMT3A* -448A > G (rs1550117) and *DNMT3B* -149C > T (rs2424913) polymorphisms, are often linked to changes in DNA methylation levels [15,16,17,18,19]. Therefore, we also investigated the contribution of these major polymorphisms of genes involved in the folate pathway to *DNMT1* promoter methylation levels.

## 2. Results

### 2.1. Distribution of the Investigated Variables in the Study Population

The demographic characteristics of the study population and the average methylation levels of the *DNMT1* gene are shown in Table 1. In total, 215 healthy Italian subjects, including 85 males and 130 females of mean age 76.4 years, composed the study cohort. Plasma Hcy, serum folate, and vitamin B12 levels were available from 158 individuals, and their average values are shown in Table 1.

**Figure 2:**
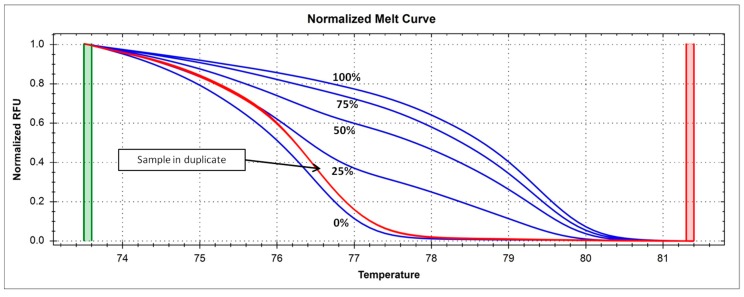
Melting curve of the *DNMT1* gene promoter showing in blue the standard samples with known methylation percentages (0%, 25%, 50%, 75%, 100%) and a sample in duplicate (red curve) showing a methylation of about 6%.

The promoter methylation levels of the *DNMT1* gene ranged from 0% to 15% in the study population (Figure 2), with an average value of 2.2% (Table 1), which is in agreement with previous investigations in other cohorts [14,20,21].

The genotype distribution of the investigated polymorphisms is shown in Table 2. Genotype frequencies are in agreement with those previously observed in healthy Caucasians [19,22], and conformed to Hardy–Weinberg equilibrium (HWE) expectations (*p* > 0.05).

### 2.2. Correlation between *DNMT1* Methylation and Biochemical Variables

**Figure 3:**
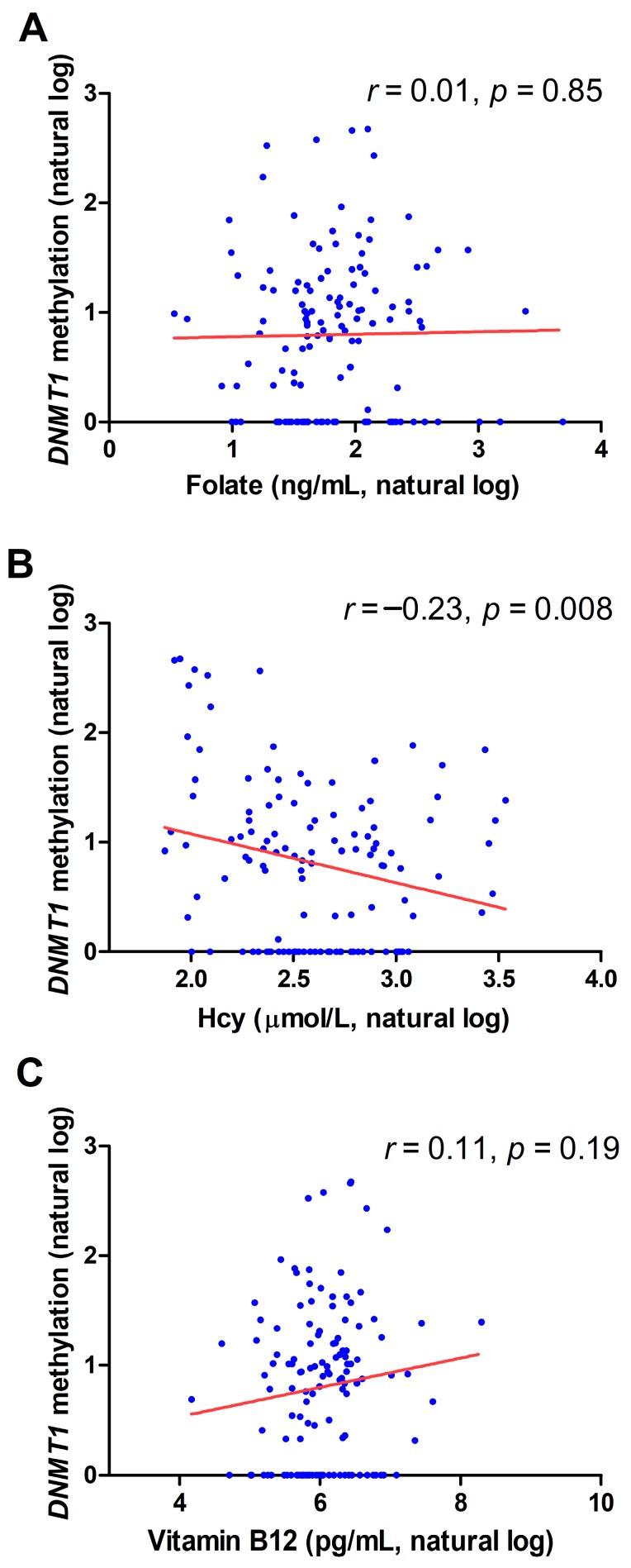
Correlation between *DNMT1* promoter methylation and circulating folate (**A**), homocysteine (Hcy) (**B**), and vitamin B12 levels (**C**).

Figure 3 shows the correlation between *DNMT1* promoter methylation and circulating folate, Hcy, and vitamin B12 levels. A significant inverse correlation between *DNMT1* promoter methylation and plasma Hcy levels was observed (*r* = −0.23, *p* = 0.008). No correlation was observed between *DNMT1* promoter methylation and serum folate levels (*r* = 0.01, *p* = 0.85), whilst a trend for correlation with serum vitamin B12 was observed, although not statistically significant (*r* = 0.11, *p* = 0.19).

### 2.3. Gender and Age Effect on *DNMT1* Methylation Levels and Biochemical Variables

**Figure 4:**
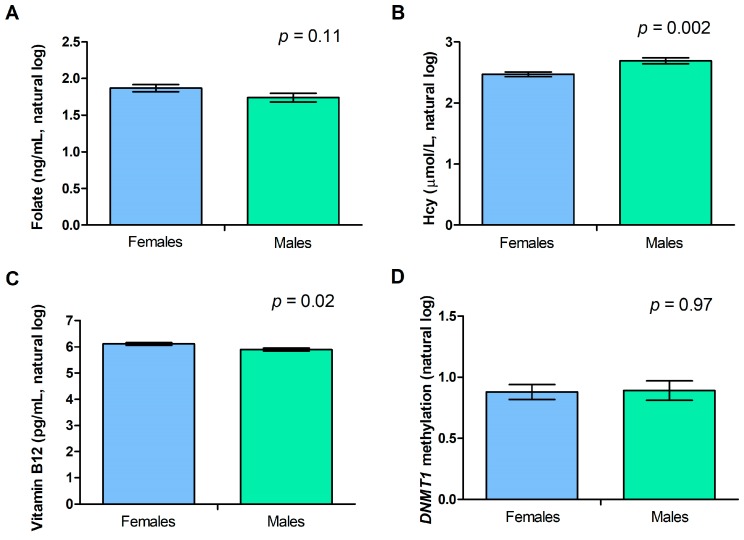
Analysis of variance showing gender differences in folate (**A**), Hcy (**B**), vitamin B12 (**C**), and *DNMT1* methylation levels (**D**). Data are expressed as means ± standard error of the mean (SEM).

Figure 4 shows the contribution of gender to circulating folate, Hcy, and vitamin B12 levels, as well as to the methylation levels of the *DNMT1* gene. We observed significantly higher Hcy levels in males than in females (*p* = 0.002), whilst vitamin B12 levels were higher in females with respect to males (*p* = 0.02), and a similar trend was observed for folates, even if the gender difference was not statistically significant (*p* = 0.11). No gender difference was observed for *DNMT1* methylation levels (*p* = 0.97) (Figure 4).

**Figure 5:**
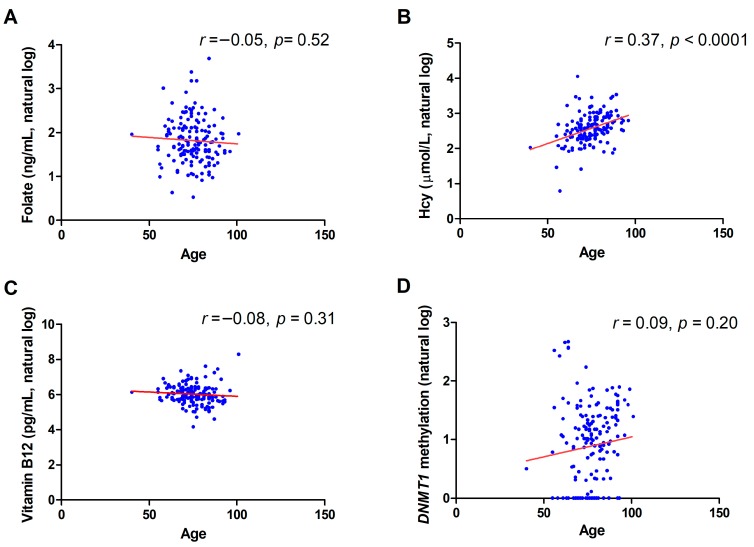
Correlation between age and circulating folate (**A**), Hcy (**B**), vitamin B12 levels (**C**), and *DNMT1* promoter methylation levels (**D**).

The contribution of age to the studied variables is shown in Figure 5. A positive correlation was observed between Hcy levels and age (*r* = 0.37, *p* < 0.0001). No significant correlation was observed between age and the other variables (folate, vitamin B12, and DNMT1 methylation levels).

### 2.4. *DNMT1* Methylation Levels and Polymorphisms of the Folate Pathway Genes

**Figure 6:**
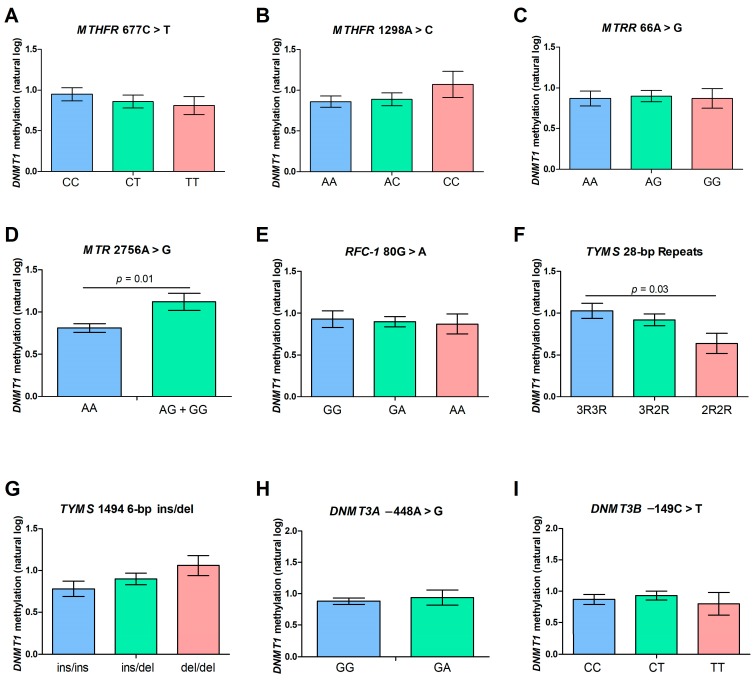
Analysis of variance (ANOVA) showing the differences in *DNMT1* methylation levels among carriers of different genotypes, adjusted for age and gender (**A**–**I**). Data are expressed as means ± SEM. Only Bonferroni’s corrected *p*-values (< 0.05) are shown. We grouped together heterozygous (AG) and homozygous (GG) *MTR* carriers (D) because only three subjects in our cohort showed the mutant *MTR* 2756GG genotype.

Analysis of variance (ANOVA) in the whole cohort of subjects revealed a statistically significant contribution of both the *MTR* 2756A > G and the *TYMS* 28-bp repeat polymorphisms to *DNMT1* promoter methylation levels. In particular, a significant increase in *DNMT1* methylation levels was observed in carriers of the *MTR* mutant allele (AG + GG) with respect to wild-type (AA) subjects (*p* = 0.01), and the *TYMS* 28-bp repeat polymorphism had a significant effect on *DNMT1* methylation levels in our cohort, with carriers of the 3R/3R genotype showing significantly higher methylation levels than 2R/2R carriers (*p* = 0.03). None of the other polymorphisms showed a significant contribution to *DNMT1* methylation levels (Figure 6). Overlapping results were observed in the subgroup of 158 subjects with available data on folate, Hcy, and vitamin B12 levels (not shown).

## 3. Discussion

In the present study, we investigated the contribution of circulating folate, Hcy, and vitamin B12, as well as of major polymorphisms of the folate pathway genes, as modulators of *DNMT1* promoter methylation levels in blood DNA from healthy individuals, observing an inter-individual variability in *DNMT1* promoter methylation levels, which ranged from 0% to 15% in the studied cohort, a significant inverse correlation with plasma Hcy levels, and a trend for a positive correlation with vitamin B12 levels. Furthermore, carriers of the *MTR* 2756G allele (AG + GG) showed a significantly higher *DNMT1* promoter methylation than non-carriers (AA), as well as carriers of the *TYMS* 28-bp repeat 3R/3R genotype with respect to 2R/2R carriers. None of the other investigated polymorphisms were linked to the methylation status of the *DNMT1* promoter. Age and gender had no significant effect on *DNMT1* promoter methylation levels in our cohort.

Present data confirm previous investigations of the methylation status of the *DNMT1* promoter in blood DNA samples, all reporting low methylation levels in average, albeit with inter-individual variability [14,20,21]. Furthermore, we confirmed previous findings obtained in a cohort of 100 Alzheimer’s disease individuals and matched controls, showing that elevated plasma Hcy levels in the AD subjects were linked to reduced methylation levels of several genes in blood DNA, including *DNMT1* [14]. Homocysteine is a metabolite for methionine production, and methionine is the precursor of SAM that serves as the universal one-carbon donor for DNA methylation reactions (Figure 1), such that hyperhomocysteinemia (HHcy) is often linked to reduced DNA methylation potential [23], and the epigenetic dysregulation of gene expression is a pathogenic consequence of HHcy in many human diseases [24]. In this context, previous in vitro studies revealed that Hcy treatment altered the expression levels of *DNMT1*, leading to global or gene-specific DNA methylation changes [25,26,27]. For example, Hcy treatment enhanced *DNMT1* expression in oocytes leading to hypermethylation of the mitochondrial DNA [25], upregulated *DNMT1* expression leading to DNA hypermethylation of the mitofusin 2 gene promoter in vascular smooth muscle cells [26], or induced DNA hypomethylation of the cyclin A promoter in endothelial progenitor cells through downregulated expression of *DNMT1* [27]. A mechanistic link between Hcy and DNA methylation is the resulting reduction of the *S*-adenosylmethionine/*S*-adenosylhomocysteine ratio following HHcy, and the subsequent accumulation of *S*-adenosylhomocysteine, which in turn inhibits methyl transfer reactions; however, it was also suggested that Hcy can alter the binding of transcription factors to the *DNMT1* promoter [25,26,27]. The present study suggests that Hcy levels correlate with *DNMT1* promoter methylation levels, thus strengthening the contribution of this metabolite to the regulation of DNA methylation.

Original findings of the present study are the correlations between *DNMT1* promoter methylation levels and both *MTR* 2756A > G and *TYMS* 28-bp repeat polymorphisms. Particularly, we observed increased *DNMT1* methylation levels in carriers of the *MTR* 2756G allele compared to the wild-type *MTR* 2756AA genotype. The *MTR* gene encodes for methionine synthase, the enzyme that catalyzes the remethylation of Hcy to methionine, and cobalamin (vitamin B12) is a cofactor in this reaction (Figure 1). The *MTR* 2756A > G polymorphism impairs *MTR* function and stability [28,29] and is often linked to either global or gene-specific DNA methylation changes in human cells and tissues [18,30,31,32,33]. For example, the *MTR* 2756A > G polymorphism was linked to increased global DNA methylation levels in human leukocytes [30], and carriers of the variant G allele had a significant increase of LINE-1 (long interspersed nuclear element 1) methylation in histologically normal breast tissues, compared to those carrying the common AA genotype [18]. Other studies reported association of this polymorphism with global methylation levels of leukocyte DNA [31], with *MTHFR* methylation levels in blood DNA of valproate-treated patients with epilepsy [32], and with hypermethylation of tumor suppressor genes in cancer specimens [33] or adjacent healthy tissues [17]. The present finding of a correlation between the *MTR* 2756A > G polymorphism and *DNMT1* promoter methylation levels in blood DNA not only strengthens the contribution of this genetic variant to the DNA methylation levels in leukocytes, but it could also be of relevance for those disorders, such as hematological malignancies, autoimmune/inflammatory disorders, and solid tumors, that are linked to impaired *DNMT1* methylation and/or expression [5,6,7,8,9,10,11,12]. For example, changes in *DNMT1* methylation levels were observed in peripheral blood DNA samples from ALL patients [8], the *MTR* 2756A > G polymorphism was linked to the risk of pediatric ALL [34], and several maternal polymorphisms of the *MTR* gene were recently proposed as responsible of aberrant methylation of ALL-related genes in their offspring [35]. Furthermore, the *MTR* 2756A > G polymorphism was also linked to the risk of autoimmune/inflammatory diseases and solid tumors, all characterized by impaired DNMT activities and global and gene-specific DNA methylation changes [36,37,38,39], suggesting that the investigation of the contribution of this polymorphism to *DNMT1* methylation levels is warranted in all these disorders.

TYMS and MTHFR compete for 5,10-methylenetetrahydrofolate in such a way that the folate pathway can be shifted from DNA methylation to the synthesis of DNA precursors, based on cellular demands (Figure 1). In this regard, the 28-bp tandem repeat polymorphism, located in the 5′UTR of the *TYMS* gene, works as enhancer for *TYMS* transcription, with higher efficiency conferred by the 3R allele with respect to the 2R one. This enhanced expression minimizes uracil misincorporation into the DNA and increases the availability of DNA precursors, such that this polymorphism is often associated with cellular growth and risk of human malignancies, including leukemias, lymphomas, breast cancer, and thoracic neoplasms, among others [40,41,42,43]. Less is known about the contribution of *TYMS* polymorphisms to the DNA methylation levels, even if the *TYMS* 28-bp tandem repeat polymorphism was associated with gene-specific methylation levels in the blood DNA of patients systemic lupus erythematosus [44], and other *TYMS* polymorphisms were linked to either global or gene-specific methylation levels in both healthy and cancerous tissues, reinforcing the evidence that DNA synthesis and methylation are interconnected pathways [17,45,46,47]. The present findings of a correlation between the *TYMS* 28-bp tandem repeat polymorphism and *DNMT1* promoter methylation levels in blood cells highlight the contribution of this gene to DNA methylation, and suggest that further investigation is warranted in human disorders associated to this variant.

The main limitation of the present study was that we investigated an elderly population of limited sample size that, despite healthy at collection, could include individuals with age-related subclinical conditions potentially impacting the findings. Therefore, results should be considered preliminary, and replication in a larger cohort and in younger subjects is warranted. Moreover, the observed *DNMT1* methylation levels were low on average, and their inter-individual variability could result from subclones of cells with high levels of *DNMT1* methylation in some individuals. Further investigations of single-cell DNA methylation levels are warranted to clarify this issue and their potential correlation with preclinical conditions in humans.

In conclusion, we investigated the contribution of both metabolites and genetic polymorphisms in the folate metabolic pathway to the *DNMT1* promoter methylation levels in blood DNA from healthy individuals, observing an inverse correlation with plasma Hcy levels, and significant associations for both *MTR* 2756A > G and *TYMS* 28-bp tandem repeat polymorphisms. The present study highlights the links existing between the folate metabolic pathway and the promoter methylation levels of the maintenance DNMT, suggesting that further studies are required to address this issue in human disorders characterized by changes in *DNMT1* promoter methylation, such as hematological malignancies, solid tumors, and autoimmune/inflammatory diseases.

## 4. Materials and Methods

### 4.1. Study Population

We collected peripheral blood samples from 215 healthy individuals, including 85 males and 130 females of mean age 76.4 ± 9.6 years (Table 1). All individuals were volunteer subjects of Italian origin, underwent a rigorous clinical and neurological examination before inclusion in the study, and were healthy at blood drawing. In addition, individuals taking drugs, substances, or vitamin supplements known or suspected to interfere with DNA methylation, such as anti-cancer drugs, anti-epileptic drugs, anti-inflammatory drugs, epi-drugs, metformin, tobacco smoking, folic acid, or other vitamins, were not enrolled in the study. Written informed consent for inclusion in the study was collected from each subject. The study was conducted in accordance with the Declaration of Helsinki and was approved by the Ethics Committee of the Pisa University Hospital (Protocol number 3618/2012).

### 4.2. Collection of DNA Samples

Genomic DNA was extracted from peripheral blood samples using the QIAamp® Blood Mini Kit (Catalog No. 51104, Qiagen, Milan, Italy) following the manufacturer’s instructions. DNA samples were stored at −20 °C until assayed.

### 4.3. Biochemical Analyses

The measurement of plasma Hcy, serum vitamin B12, and folate levels was performed with the standard diagnostic laboratory methodologies of the Pisa University Hospital, as detailed elsewhere [48].

### 4.4. Genotyping for Common Polymorphisms in Genes of the Folate Metabolic Pathway

PCR/RFLP techniques, detailed elsewhere [49,50], were applied for genotyping all the study polymorphisms, namely, *MTHFR* 677C > T (rs1801133) and 1298A > C (rs1801131), *MTR* 2756A > G (rs1805087), *MTRR* 66A > G (rs1801394), *SLC19A1* (*RFC-1*) 80G > A (rs1051266), *TYMS* 28-bp repeats (rs34743033) and 1494 6-bp indel (rs34489327), *DNMT3A* −448A > G (rs1550117), and *DNMT3B* −149C > T (rs2424913).

### 4.5. Analysis of DNMT1 Methylation Levels

In total, 200 ng of DNA from each sample was quantified using a Nanodrop ND 2000c spectrophotometer (NanoDrop, Thermo Scientific, Wilmington, DE, USA). The DNA was subsequently treated with sodium bisulfite, using the EpiTect Bisulfite Kit (Catalog No. 59104, Qiagen, Milan, Italy), to convert unmethylated cytosines into uracil. All the samples were treated simultaneously in order to avoid potential batch effects. The bisulfite conversion efficiency, assessed using a sample of completely unmethylated human DNA (Catalog No. 59568, Qiagen, Milan, Italy), was 99% on average. For the analysis of *DNMT1* methylation levels, we applied the methylation-sensitive high-resolution melting (MS-HRM) technique, using a protocol previously developed, validated, and fully described by us elsewhere [51]. All the analyses were performed in a CFX96 Real-Time PCR detection system (Bio-Rad, Milan, Italy). We investigated a CpG island in the promoter/5′UTR region of the *DNMT1* gene, whose methylation levels were previously linked to *DNMT1* gene expression levels [5,8]. Table 3 lists the sequence of the primers and the details of the investigated region. Each reaction was performed in duplicate, and 10% of the samples were analyzed independently on separate occasions to verify the inter-assay variability. The mean standard error for inter-assay variability was 0.1%, indicating a very good technical reproducibility. We mixed fully methylated and unmethylated DNA (EpiTectH methylated and unmethylated human control DNA, bisulfite-converted (Catalog No. 59695, Qiagen, Milan, Italy), to obtain the following ratios of methylation: 0%, 25%, 50%, 75%, and 100%. These standard DNA samples were included in each assay to generate standard curves (Figure 2) that were used to deduce the methylation levels of each sample, as detailed elsewhere [52].

### 4.6. Statistical Analyses

The chi-square (Χ^2^) analysis was used to investigate deviations from Hardy–Weinberg equilibrium. Linear regression analysis was used to investigate the correlation between *DNMT1* promoter methylation levels and plasma Hcy, serum folate, or vitamin B12 levels. Similarly, linear regression was applied to investigate the correlation of these variables with age. Analysis of variance (ANOVA) was used to evaluate gender differences in *DNMT1* promoter methylation levels and plasma Hcy, serum folate, or vitamin B12 levels. ANOVA, including age at sampling and gender as covariates (and also plasma Hcy, serum folate, and vitamin B12 levels in the subgroup of patients with available metabolites), was used to investigate the contribution of each of the studied polymorphisms to *DNMT1* methylation levels, followed by post hoc Bonferroni’s correction for multiple testing. The Shapiro–Wilk test was used to check for normality, and natural logarithm transformation of data that did not follow a normal distribution (*DNMT1* methylation levels, plasma Hcy, serum folate, and vitamin B12) was done before the analyses. Statistical analyses were performed with the STATGRAPHICS 5.1 plus software package for Windows and the MedCalc statistical software v. 12.5. We considered Bonferroni’s corrected *p*-values < 0.05 as statistically significant.

## Figures and Tables

**Table 1:** Study population.

Number of Subjects	Age (Years) Mean ± SD	Gender	Folate * (ng/mL) Mean ± SD (SEM)	Hcy * (μmol/L) Mean ± SD (SEM)	Vitamin B12 * (pg/mL) Mean ± SD (SEM)	*DNMT1* Methylation (%) Mean ± SD (SEM)
Total: *N* = 215	76.4 ± 9.6	M: 85 F: 130	-	-	-	2.2 ± 2.5 (SEM = 0.2)
Subgroup with biochemical data: N = 158	74.9 ± 9.3	M: 69 F: 89	7.16 ± 4.86 (SEM = 0.39)	14.36 ± 7.1 (SEM = 0.56)	495.6 ± 404.3 (SEM = 32.3)	2.1 ± 2.7 (SEM = 0.2)

* Data available from the subgroup of 158 individuals. M—male; F—female; SEM—standard error of the mean; Hcy—homocysteine.

**Table 2:** Distribution of genotypes in the study population; indel—insertion/deletion.

Polymorphism	Genotypes: *N* of Subjects (%)
*MTHFR* 677C > T	CC: 77 (35.8%), CT: 94 (43.7%), TT: 44 (20.5%)
*MTHFR* 1298A > C	AA: 101 (47%), AC: 92 (42.8%), CC: 22 (10.2%)
*MTRR* 66A > G	AA: 63 (29.3%), AG: 116 (54.0%), GG: 36 (16.7%)
*MTR* 2756A > G	AA: 164 (76.3%), AG: 48 (22.3 %), GG: 3 (1.4%)
*RFC-1* 80G > A	GG: 60 (27.9%), GA: 119 (55.3%), AA: 36 (16.8%)
*TYMS* 28-bp Repeats	3R3R: 60 (27.9%), 3R2R: 116 (53.9%), 2R2R: 39 (18.2%)
*TYMS* 1494 6-bp indel	in/in: 67 (31.2%), in/del: 114 (53.0%), del/del: 34 (15.8%)
*DNMT3A* -448A > G	GG: 173 (80.4%), GA: 42 (19.6%), AA: 0 (0.0%)
*DNMT3B* -149C > T	CC: 89 (41.4%), CT: 107 (49.7%), TT: 19 (8.9%)

**Table 3:** Primers and annealing temperature (*T*_a_) used during methylation-sensitive high-resolution melting (MS-HRM) analysis, as well as amplicon length, region analyzed respect to the Transcription Start Site (TSS), number of cytosine–phosphate–guanine (CpG) sites, accession number, and nucleotide position of the *DNMT1* region analyzed. F—forward; R—reverse.

Primer Sequences	*T*_a_	Amplicon Length	Region Respect to TSS	CpG Sites	Accession Number and Nucleotide Position
F: 5′–GGTATCGTGTTTATTTTTTAGTAA–3′ R: 5′–ACGAAACCAACCATACCCAA–3′	52 °C	114 bp	From −106 to +8	9	NG_028016.3 41101–41215
